# Reconstructive Challenges of Distal Tibia Bone Tumors: Extracorporeally Irradiated Autograft Combined with a Nonvascularized Autograft Fibula for Superior Reconstruction and Functional Outcomes When Compared to Ipsilateral Pedicled Fibula Transfer Alone

**DOI:** 10.1155/2021/6624550

**Published:** 2021-03-23

**Authors:** Manit K. Gundavda, Manish G. Agarwal, Rajeev Reddy, Ashik Bary

**Affiliations:** Hinduja Hospital and Medical Research Centre, Mumbai, India

## Abstract

**Introduction:**

Traditionally, centralization of the fibula with fusion across the tibiotalar joint has been used to reconstruct distal tibial defects. Although effective, it requires long periods of protected weight-bearing. The fibula or the fixation often fails before fibular hypertrophy necessitating multiple additional surgeries. A method of using ECRT with the available ipsilateral fibula (nonvascularized) to reconstruct the distal tibia defect with the aim of early return to weight-bearing was evolved. This paper documents our early experience. *Patients and Methods*. Four patients; with the diagnosis of osteosarcoma in 3 patients and recurrent giant cell tumor of the bone in 1 patient, underwent resection of the distal tibia for tumors between 2017 and 2019. Extracorporeally irradiated (50 Gy) distal tibia along with ipsilateral nonvascularized fibula was used to bridge the defect and fuse the tibiotalar joint. A plate was used to rigidly hold the construct. The final outcome was compared to the historical control group that underwent only pedicled ipsilateral fibula transposition and ankle arthrodesis without recycled autograft or allograft between 2009 and 2017. Oncological reconstruction and functional outcomes were compared for each group. Patient reported outcomes on the acceptability of ankle fusion; cosmesis and function were analyzed and compared between the two groups.

**Results:**

The mean resection length in the study group (4 patients) was 7.75 cm (7 to 8.5 cm). As compared to the historical cohort of 7 patients, the study population showed statistically superior results in all reconstruction, functional, and patient-reported outcomes except time to proximal junction union (*p*=0.068). There were no reconstruction failures, infection, or nonunions in the study group, whereas the control comparative group had 2 proximal junction nonunions and a mean time to fibular hypertrophy of 143 weeks (82 to 430 weeks) with fibula centralization. Earlier weight-bearing was allowed (mean 26.75 weeks; median 27 weeks) compared to (mean 80.75 weeks; median 80 weeks) in the control group.

**Conclusion:**

We think that ECRT with ipsilateral vascularized fibula is a promising method of reconstructing the distal tibia. The recycled autograft tibia added strength to the distal tibia construct in our study and aided the anatomical reconstruction of the distal tibia. The patient-reported outcomes for cosmesis and acceptability add to the benefits of performing this procedure. Consistent early union across the proximal junction and earlier weight-bearing were clear advantages of this method.

## 1. Introduction

**Figure 1:**
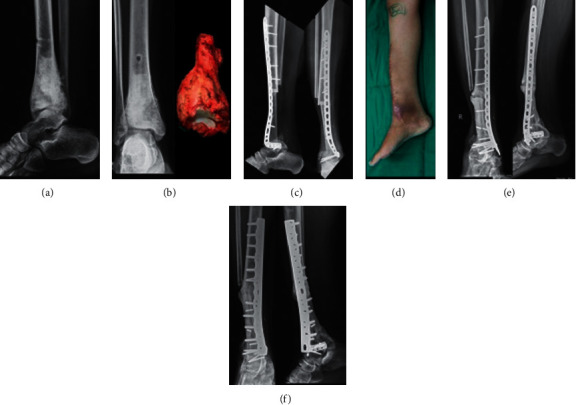
A 13-year-old female presented with a biopsy-proven distal tibia osteosarcoma (a), and following neoadjuvant chemotherapy, she underwent resection of the tumor (b) and reconstruction with centralization of ipsilateral fibula as a vascular graft (c). The midline incision was extended medially for plating across the ankle (d), and this posed a challenge with inadequate soft tissue cover over the reconstruction and led to a sinus formation. Plate breakage was observed at 39 months along with fibular hypertrophy and nonunion across the proximal junction (e), and this required bone grafting and plate exchange (f) to achieve union.

Distal tibia is an uncommon location for primary malignant tumors of the bone. Most resections include the articular surface, and reconstructions of these defects pose unique challenges including a complex bony anatomy and a thin soft tissue cover. Unlike around the knee or hip, there seems to be no reliable endoprosthetic or biological reconstruction for creating a functional joint at the ankle [1–7]. A below-knee amputation can give excellent functional outcomes and is therefore a suitable alternative to any reconstruction that fuses the tibiotalar joint [8, 9]. The centralization of the fibula (Figures 1(a)–1(c)) into the defect as a vascularized autograft has been used for many years, but the fibula takes a long time to hypertrophy requiring protracted protected weight-bearing (Figure 1(d)) [1, 8, 10]. Additionally, this long period risks breakage of the fixation implants and fractures of the fibula (Figure 1(e)) which many a times require repeated surgical procedures (Figure 1(f)). Inspired from long diaphyseal defect reconstructions, we combined the extracorporeally irradiated tibia after tumor resection with the ipsilateral nonvascularized fibula with an ankle fusion. The extracorporeally irradiated tibia provides the strength till the fibula incorporates, aids union, and gains strength. This paper presents our early results from four such cases.

## 2. Patients and Methods

**Figure 2:**
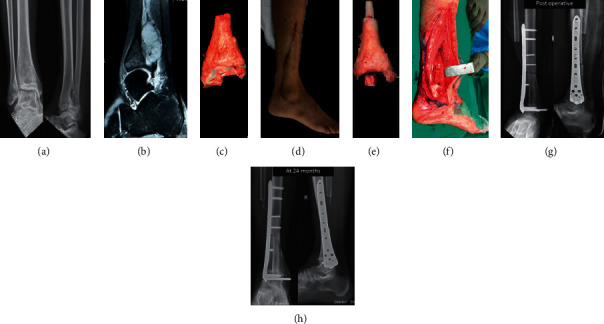
A 16-year-old male presented with a radiograph (a) and MRI (b) suggestive of a distal tibia osteosarcoma that was confirmed on a core needle biopsy. The distal tibia resection was performed with a bone margin of 2 cm and a soft tissue margin of healthy cover over the tumor (c). Procedure was performed through the anterolateral approach to allow tumor resection, fibula to be harvested, and adequate muscle cover over the lateral fixation after implantation (d). Following extracorporeal irradiation, the distal tibia tumor bone was prepared for reimplantation and the ipsilateral nonvascular fibula was inserted intramedullary spanning across both junctions (e). The construct was placed into the defect after the talus dome was prepared to achieve bony surface (f). After the fibula is confirmed to be across the proximal and distal junctions, a locking plate was used for fixation (g). Healed proximal osteotomy junction and fused ankle arthrodesis junctions with the fibula healing and incorporation seen on the latest follow-up radiograph (h).

Four patients with primary bone tumors of the distal tibia (3 osteosarcoma and 1 recurrent giant cell tumor (GCT)) underwent limb salvage surgery following appropriate neoadjuvant therapy (chemotherapy for osteosarcoma and denosumab for GCT). Resection margins were planned on pretreatment radiographs and magnetic resonance imaging scans (Figures 2(a) and 2(b)) to achieve a 2-3 cm bony margin and soft tissue cover as margin over the resected specimen (Figure 2(c)). Patients were positioned in supine position, and incision was placed anterolaterally to include the biopsy tract that was excised along with the tumor (Figure 2(d)). The anterolateral incision was proposed to additionally aid in the ipsilateral fibula harvest and allow lateral plating for reconstruction and arthrodesis.

The resected tumor bone is given a single fraction of 50 Gy radiation using a linear accelerator over 20 to 25 minutes as per our usual protocol [11]. The radiated tibial segment is stripped of all soft tissue, and the intramedullary canal is reamed to appropriate size to allow the fibula graft to be inlayed (Figure 2(e)). The talus dome and distal tibial cartilage are burred to expose the subchondral bone at both ends for ankle arthrodesis (Figure 2(f)). The fibula is allowed to telescope across the proximal and distal junctions before fixation with a lateral locking plate (Figure 2(g)).

Postoperative rehabilitation allowed immediate mobilization without loading the operated limb. Partial weight-bearing was started at 3 months, progressing to full weight-bearing at nearly 6 months, once union was observed. Follow-up was at 3-month interval for assessment of oncological and reconstruction outcomes. Orthogonal radiographs were performed at each follow-up to assess union as bridging callus or disappearance of osteotomy junction in at least three out of the four cortices (Figure 2(h)), and confirmation of union was required before allowing full weight-bearing. Functional outcomes were recorded only after junction union and return to full weight-bearing mobilization.

The comparative group comprised all patients that underwent distal tibia resections for primary bone tumors and reconstruction with fibula centralization between 2010 and 2017. Their surgical details were available from medical records and functional and reconstruction outcomes documented at each follow-up visit. Fibular hypertrophy was defined as 50% increase in the transposed fibular dimension as noted on orthogonal anteroposterior and lateral radiographs. Both groups were analyzed for functional (MSTS and AOFAS ankle and hindfoot scores) and reconstruction outcomes (bony union, reconstruction failure, and time to weight-bearing) as well as patient-reported outcomes on acceptability of ankle arthrodesis and cosmesis using a Likert's scale.

Data were analyzed for descriptive statistics, and the Mann–Whitney “U” test was applied to assess significance with two-tailed *p* value < 0.05 using SPSS Version 25 (Chicago, IL, USA).

The institution waived approval for the human protocol for this investigation, and all investigations were conducted in conformity with ethical principles of research.

## 3. Results

Two males and two females with a median age of 16.5 years (mean age: 17.75 years, range 5 to 33 years) underwent the modified procedure with extracorporeal radiation of tumor bone and reimplantation with ipsilateral fibula graft and ankle arthrodesis between 2017 and 2019. The mean resection length was 7.75 cm (7 to 8.5 cm). The comparative group consisted of 7 patients with a median age of 22 years (mean: 25.29 years, range 18 to 39 years). While all 7 patients were assessed for reconstruction outcomes, only 6 patients were available at latest follow-up for functional evaluation. 1 patient had died of metastatic disease. The study population showed statistical superior results (*p* < 0.05) in all outcomes except time for proximal junction union which was also shorter in the study group but not reaching statistical significance (*p*=0.068) (Table 1). Reconstruction outcomes were superior in the study group for ankle joint union (*p*=0.036) and time to initiation of weight-bearing (*p*=0.019) and full weight-bearing mobilization (*p*=0.011).

Functional scores were significantly superior in the study population: MSTS mean 28.25 (*p*=0.0003) and AOFAS mean 83/100 (*p* < 0.0001). Patient-reported outcomes for acceptability and cosmesis too were significantly superior in the study population (*p*=0.0008).

There were no reconstruction failures, infection, or nonunions in the study group at the latest follow-up (mean follow-up: 81 weeks; range 52 to 112 weeks). The comparative group had 2 out of the 7 proximal junction nonunions that were managed with refixation and bone grafting. Both these patients presented with hypertrophic nonunion and implant failure (plate breakages). 6 of the 7 patients had implant failures, 2 plate breakages that required revision, and 4 patients with screw fractures but did not require revision of implantation. Average time to fibular hypertrophy was 143 weeks (82 to 430 weeks) at a mean follow-up of 8.16 years (3.5 to 10.5 years) in the comparative group.

## 4. Discussion

Reconstruction following resection of distal tibia for primary bone tumors poses a challenge to the orthopedic surgeon. Although below-knee amputations for distal tibia tumors may provide excellent function with modern prostheses, limb salvage continues to be offered as a standard of care in view of psychological impact and quality of life [8, 9]. A variety of procedures have been described in the literature to achieve limb salvage; however, each of them are associated with their inherent problems, namely [7], delay in weight-bearing and graft failures seen with autogenous bone grafting [12, 13] as well as fibular autograft and arthrodesis [1, 8, 10, 14]; infection and graft subsidence/lysis concerned with osteoarticular allografts [15–17]; distraction osteogenesis [2, 14]; and also endoprosthetic reconstruction [3, 18–20] failing with infection or inadequate soft tissue coverage. The best option however remains debatable. Use of the ipsilateral fibula alone or augmented with auto/allograft for gap reconstruction remains the most common method [5], and centralization of the fibula with ankle arthrodesis has been routinely performed at our institute for distal tibial tumors as it offers an easy, inexpensive option for reconstruction [8].

Restoration of the functional ankle joint has remained a challenge following distal tibia resections for bone tumors [5, 7]. Biological reconstruction allows restoration of the bone in the defect but requires a fusion across the tibiotalar joint. Sambri et al. [7] demonstrated ankle joint reconstruction using osteoarticular allografts but reported osteoarthritis following biological joint reconstruction in 3 of their 11 patients. However, the symptomatic and functional worsening was not associated with the degree of osteoarthritis and is a promising procedure if ankle stability can be established [7]. Complex anatomy, deficient soft tissue, and forces of biomechanics at the ankle joint add significant stress to the construct [12, 21, 22]. Supported with evidence from the literature, we note that biological reconstructions tend to fail in nonunion causing fatigue breakage of the bone graft and/or the fixation constructs [1, 4, 7, 23]. Isolated use of fibulae or allograft has been described for many years [1, 6, 8, 10, 14, 24], and addition of complex microsurgery for vascularized bone transfer to improve outcomes have been described too [25]. Even then, these procedures continue to have high failure rates [5]. Restoration of a functional ankle joint using custom or modular mega-endoprostheses comes with its' inherent complications of loosening or infection [3, 18–20]. Distraction osteogenesis is not advocated in the setting of malignant bone tumors requiring adjuvant therapy [2].

Fibular centralization allows a vascularized fibula to be transposed into the defect with an aim of having accelerated union and hypertrophy over time to achieve “tibialisation.” This technique has its own challenges. If plate is used for fixation to hold the construct as we have done, the fibula takes a long time to hypertrophy [1, 6, 8]. If minimal fixation with *k* wires and an external cast is used with the aim of stimulating faster hypertrophy, fracture of the fibula is common and without a rigid fixation, deformity or nonunion can happen [5, 8]. The plate fixation itself is a challenge as only talus is available for fixation considering that the subtalar mobility is to be preserved. The regular precontoured plates available to fix distal tibia fractures do not fit well when extended across the ankle to the talus. Custom stainless steel “T” plates used by us have also broken in 2/7 patients when used with fibula centralization with screw breakage in 4/7 patients. The time to initiation of weight-bearing and union at the ankle joint in our experience was 38.8 weeks (median 32 weeks) and 80.57 weeks (median 80 weeks) in our two groups, respectively.

Capanna [26] described the use of an allograft combined with a vascularized fibula for long-length femur defects. The allograft added strength to the construct, and the vascularized fibula accelerated the union across the junctions which was delayed when allograft alone was used. We have been using ECRT instead of allografts over the last 15 years [10, 22]. We have used the same principles in our femur diaphyseal reconstructions with ECRT and a vascularized fibula. In the distal tibia, the defect is generally less than 10 cm, and therefore, a nonvascularized fibula coupled with ECRT distal tibia seemed like a reasonable construct. We planned access from the lateral side for easy harvest of the ipsilateral fibula. Even if the distal fibula required en-bloc resection with the tibia, enough length was available proximally in all cases. Rest of the procedure was similar where the articular surfaces of the ankle were debrided of cartilage. The fibula was passed into the medullary canal of the ECRT tibia and proximally into the tibial canal and distally into the talar body for a centimeter on either side. A slot was made in the center of talar dome to telescope the fibula across. The ECRT bone is size matched, so there is no limb-length discrepancy. Availability of size-matched recycled autograft has advantages over allografts for reconstruction ease with minimal risk of graft rejection or transmitted infections [27]. This method can be used universally since it does not require either a specialized tissue bank or any microsurgical expertise.

We achieved union across all junctions, probably attributed to the telescoping of fibula across the proximal and distal junctions. We also allowed our patients for unrestricted weight-bearing at a mean of 26.75 weeks (median 27 weeks; range 17 to 36 weeks), much earlier than the previously operated patient cohort with fibula centralization, where we waited until fibula hypertrophy was noted. Improved functional scores and patient-reported outcomes for cosmesis and acceptability add to the benefits for performing this procedure.

Although the follow-up is short, the end point of the study, i.e., evidence of union across both junctions and return to full weight-bearing; was achieved in all 4 study patients. Early results from this small series are promising; however, longer follow-up will be necessary before we can ascertain this procedure is the ideal reconstruction option following distal tibia resections. Leg-length discrepancy in 1 patient (age 5 years) where the distal physis was resected needs to be assessed at longer follow-up, and we are hopeful for acceptable outcomes as the distal tibial physis does not contribute majorly to the leg length [25]; possibility of some compensation from the proximal physis is yet to be assessed. We did observe a translation of the talus laterally as the center of the talus had to match with the medullary canal of the distal tibia for fibular telescoping. We do not know how this will impact the function of the foot in the long term. Although we cannot attribute this directly to our procedure, all our four patients have regained some dorsiflexion and plantar flexion movement from the other joints of the foot.

Use of CT scan to generate a 3D simulation of the surgery and custom precontoured locking plate for accurate positioning and arthrodesis of recycled autograft with the central axis of the talus was attempted in two patients. The first patient was a five-year-old girl requiring resection of the distal tibia for an osteosarcoma; the complexity in achieving fixation in the talus of this girl required planning and custom implantation. The second patient was a recurrent giant cell tumor in a 33-year-old female with a history of previous surgeries, gross expansion, and distortion of the ankle anatomy. She received neoadjuvant denosumab and resection followed by ECRT and fixation with a custom 3D-printed locking plate. The technique was applied in both these patients in view of complex reconstruction and additionally for further improvement of the surgical technique in terms of accuracy and reproducibility. In both these cases, we felt that the reconstruction was made easier by the customized plate.

To conclude, recycled autograft tibia added to the structural construct in our study and aided anatomical reconstruction of the distal tibia. Junction healing proximally and ankle arthrodesis were seen in all cases and probably attributed to the structural stability from the tibia and telescoping of autograft fibula crossing across both junctions. Early weight-bearing was allowed as soon as junctions healed, as early as 8 weeks, as we did not have to wait for fibula hypertrophy in presence of the distal tibial autograft. No graft, implant, or reconstruction failure was seen in our small series with early results.

## Figures and Tables

**Table 1:** Results.

	Study group, *n* = 4	Control group, *n* = 7	*p* value
Proximal junction union	28.25 weeks	93 weeks	0.068
Ankle joint union	17 weeks	47 weeks	0.036
Time to initiate weight-bearing	13.75 weeks	38.8 weeks	0.019
Time to full weight-bearing	26.75 weeks	80.57 weeks	0.011
Fibula hypertrophy	NA	143 weeks	
MSTS score	28.25	23.67	0.0003
AOFAS score	83/100	63/100	<0.0001
Patient-reported outcomes (acceptability of ankle arthrodesis and cosmesis)			
Likert's scale	4.5/5	3.2/5	0.008

## Data Availability

Data are available upon request to the corresponding author.
